# The Use of Selfie Camera Traps to Estimate Home Range and Movement Patterns of Small Mammals in a Fragmented Landscape

**DOI:** 10.3390/ani12070912

**Published:** 2022-04-02

**Authors:** Ana Gracanin, Katarina M. Mikac

**Affiliations:** Centre for Sustainable Ecosystem Solutions, School of Earth, Atmospheric and Life Sciences, Faculty of Science, Medicine and Health, University of Wollongong, Wollongong, NSW 2522, Australia; anagracanin@outlook.com

**Keywords:** camera trap, home range, overlap, fragmentation, movement

## Abstract

**Simple Summary:**

Camera trapping allows scientists to study a range of species, across large areas for long periods of time, with little impact on animals. It has been readily used to study movements and territories of large mammals but not for smaller species. This is because they often appear too small on camera to be able to identify distinct individuals. The selfie trap is a method that allows for up close and detailed images of small mammals, and our study aimed to test the ability of the method to track the movement and social behaviour of sugar gliders. We found that the selfie trap is an efficient camera trapping method for estimating home ranges and movements due to its ability to obtain high recapture rates for multiple small mammal species and individuals. In our study landscape, linear strips of habitat were readily utilised by all small mammals, highlighting their importance as wildlife corridors in a fragmented landscape.

**Abstract:**

The use of camera traps to track individual mammals to estimate home range and movement patterns, has not been previously applied to small mammal species. Our aim was to evaluate the use of camera trapping, using the selfie trap method, to record movements of small mammals within and between fragments of habitat. In a fragmented landscape, 164 cameras were set up across four survey areas, with cameras left to record continuously for 28 nights. Live trapping was performed prior to ear mark animals to facilitate individual identification on camera. Four small mammal species (sugar glider; *Petaurus breviceps*; brown antechinus; *Antechinus stuartii*, bush rat; *Rattus fuscipes*, and brown rat; *Rattus norvigecus*) were recorded on camera (N = 284 individuals). The maximum distance travelled by an individual sugar glider was 14.66 km, antechinus 4.24 km; bush rat 1.90 km and brown rat 1.28 km. Movements of both female and male sugar gliders in linear fragments were recorded at much higher rates than in larger patches of forest sampled in grids. Short term core homes ranges (50% KDE) of 34 sugar gliders ranged from 0.3 ha to 4.2 ha. Sugar glider core home ranges were on average 1.2 ha (±0.17) for females and 2.4 ha (±0.28) for males. The selfie trap is an efficient camera trapping method for estimating home ranges and movements due to its ability to obtain high recapture rates for multiple species and individuals. In our study landscape, linear strips of habitat were readily utilised by all small mammals, highlighting their importance as wildlife corridors in a fragmented landscape.

## 1. Introduction

The study of animal movements allows ecologists to understand species’ behaviours related to: habitat selection, territoriality, foraging, mating, and dispersal, as well as investigate responses to anthropogenic changes in the environment [1,2,3]. Barriers to animal movement such as habitat fragmentation and roads, have received significant attention because of the conservation concerns around population viability from restricted movement across a landscape [4,5,6,7].

Most noticeable is the impact of fragmentation on arboreal species as they rely on continuous canopy, otherwise populations can decline due to the isolation of available habitat patches [8,9,10,11]. Fragmentation can result in changes in home range behaviour, such as where individual animals will have smaller home ranges and restrict their movements [12]. When in fragmented landscapes, Langurs (*Semnopithecus* spp.), a mostly arboreal primate, were found to adopt an energy conservation strategy were individuals spent more time feeding than moving, resulting in a smaller home range and diets shifting to consume different plant species [12]. However, other species may not adopt similar energy conservation strategies due to the disjointed distribution of resources within the fragmented landscape. Male brushtail possums (*Trichosurus vulpecula*) were found to travel further than females in linear strips of roadside habitat [13]. It was found that both sexes had greater levels of activity in roadside habitat compared to other forest fragments, and this indicates that linear shaped strips of habitat could be considered as energetically challenging as ranging behaviours must increase to access resources [13]. Therefore, the movement behaviour of arboreal mammals within a fragmented landscape can change adaptively [12] or remain energetically costly as it will depend on species resource requirements (e.g. males seeking mates or dispersing) and the spatial availability of these resources within the landscape [13]. In the case of arboreal marsupials, recent observations of species normally perceived as strictly arboreal, have been documented moving on the ground [14,15]. Whether these observations reflect the potential for sensitive arboreal species to disperse through the matrix (rather than be solely reliant on tree cover), is yet to be determined.

To study movements and home ranges a variety of methods to track individual animals are available to ecologists. Many home range studies utilise tracking collars [16,17,18] or directly resight tagged individuals over a study period [19,20,21]. However, these methods can be costly, difficult or have impacts on animal welfare. Since the advent of camera trapping methods, that allow for highly effective and efficient surveys of wildlife, the method has also had successful application in estimating home ranges of many mammal species [22,23,24,25,26]. However, to date, all home range studies using motion sensor cameras, have been focused on large charismatic mammals, particularly felines with unique fur patterns [27,28,29].

The use of camera traps to estimate home range and track movements of small mammal species has not been previously undertaken. In the context of small mammals, cameras have limited focus ranges resulting in low resolution images that can then lead to issues in correct species identification. Some researchers have modified camera placement, for example mounting cameras to face down, parallel with the ground, to obtain images in higher clarity [30,31,32] or placed in protective housing for intertidal areas [33]. More recently, purpose-designed camera traps have been made to obtain facial images of small mammal species, a method aptly named the selfie trap [34]. Through several modifications the selfie trap is able to record small mammals in high resolution for accurate species and individual identification [34]. Many small mammal movement studies use intensive repeated live trapping surveys for mark-recapture (e.g. 12,171 live trapping nights [35]) and radio-tracking very small animals is highly difficult due to size limitations and battery life [36,37,38]. Thus, replacing live trapping mark-recapture with camera trapping would be more efficient, as recapture rates are higher for camera traps as they are considered an ‘open’ trap [39]. One study compared the use of live trapping grids to estimate sugar glider (*Petaurus breviceps*) home range, and when calibrated with radio-telemetry data, found the use of recaptures through live trapping to be a valid method for home range estimation [40]. As live trapping is an intensive process, the use of camera trapping for small mammals would be more effective for estimating movements and home ranges. 

The focus of our study was to understand if selfie traps, a modified camera trapping method that targets small mammals, could be used to track their movements, and calculate home range. The target species of this study was the sugar glider (*P. breviceps*), with opportunistic data collected on other small mammals (brown antechinus; *Antechinus stuartii*, bush rat; *Rattus fuscipes*, and brown rat; *Rattus norvigecus*). We aimed to compare short term movements of small mammals between linear patches of habitat compared with larger patches of habitat. We aimed to estimate short term core home ranges of sugar gliders and make observations on their spatial and social organisation in a fragmented landscape. 

## 2. Materials and Methods

### 2.1. Study Area

**Figure 1:**
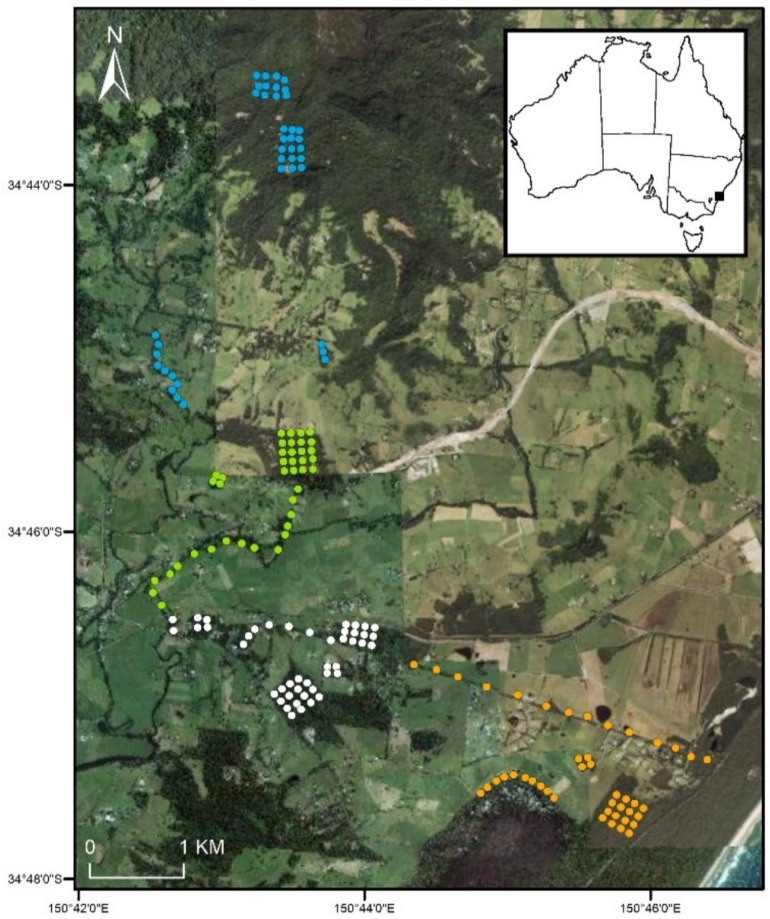
Site locations in the landscape near Berry, NSW (n = 164). Each site had a live trap (Elliott A trap) for seven nights and this was immediately replaced with a selfie trap for 28 nights. Areas one (orange), two (white), three (green) and four (blue) were surveyed immediately after each other sequentially (August to November 2019). The number of sites in each area was: Area one = 44, Area two = 45, Area three = 40, and Area four = 40.

This study was part of a broader project investigating arboreal marsupial abundance and distribution within a fragmented landscape around the township of Berry, located approximately 140 km south of Sydney, New South Wales, Australia (Figure 1). The study design focused on four major areas of interest as part of research into the Berry wildlife corridor [41,42]. Vegetation towards the coast (16 m a.s.l.) was a combination of littoral rainforest, and blackbutt-dominated (*Eucalyptus pilularis*) forest [14]. East of the coast, majority of the fragments of available habitat was either blackbutt-dominated or turpentine-dominated (*Syncarpia glomulifera*) forests. The creek lines in the study area mostly contained *Casuarina sp.* Sites furthest to the north on the Illawarra Escarpment (410 m a.s.l.), contained sub-tropical rainforest. The fragments south of the escarpment were surrounded by agricultural land, majority of which were pastures of grass for cattle and other livestock. At the nearest weather station, Kiama Bombo Headland, annual rainfall was 646.0 mm and mean monthly temperatures ranged between 25.4 °C (February) and 10.1 °C (July) [43]. 

### 2.2. Live Trapping

The study area was divided into four survey areas that targeted key areas of the Berry corridor with 164 trap sites in total (Figure 1). Each area survey was performed for four weeks (28 camera trap nights), with area one live trapping starting on the 13 August 2019, before sequentially moving onto the next area. Each area was surveyed in distinct linear transects, or grids, which was dependent on the type of fragment of habitat being surveyed (Figure 1). The focus species of the study was the sugar glider and traps were set 100 m apart to study sugar glider ecology [40]. Traps were spaced 100 m apart in grids [40], and an average of 250m along linear transects (dependent on private property limitations). This spacing of traps did not appropriately target the study of movements of other species, such as brown antechinus and bush rats, thus the data collected on these species is opportunistic (for example a spacing of 20 m between traps is more appropriate for antechinus species [35]). 

**Figure 2:**
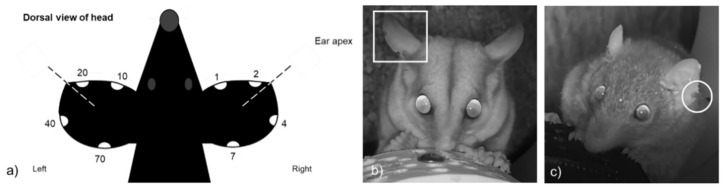
For small mammals captured, a unique code that corresponded with ear notching, was assigned (**a**). The appearance of these unique ear codes on selfie trap footage is shown in two examples: (**b**) a female sugar glider (*Petaurus breviceps*) is shown with the code ‘03’ applied (ear notch 1 and 2); and (**c**) a female antechinus (*Antechinus stuartii*) is shown with code ‘40’ applied (ear notch 40).

Elliott A traps [44] were placed up trees, secured onto platforms, at heights of 2 m above ground to target arboreal sugar gliders. Trap entrances faced the tree and peanut butter, honey and oats were used as bait [45,46]. A honey water mixture was sprayed up and down the tree that the trap was secured to and surrounding foliage [47]. Traps were checked for seven mornings before they were packed down. Animals caught had a 2 mm clipping taken on the ear margin for genetic sampling [48,49]. Each individual animal was given a unique code based on the position of the clipping (Figure 2). This allowed for recaptures of individuals to be recorded through both live trapping, and on camera footage.

### 2.3. Camera Trapping

**Figure 3:**
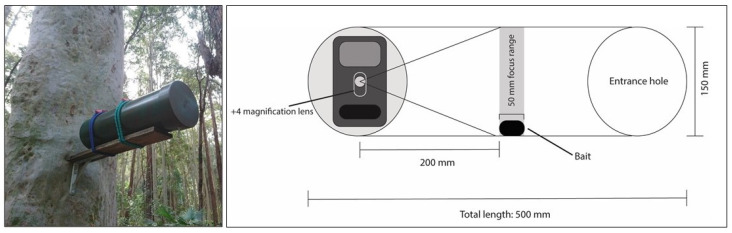
Example of a selfie trap positioned 2m off the ground to target arboreal species. Inside is a camera with an altered focal distance for close recording of small mammal species.

Following the last morning of live trapping at each area, a selfie trap (Figure 3) was placed on the same platform as the Elliott trap and left to record for four weeks (N = 164 selfie traps). The bait holders inside the selfie traps limited direct access for small mammals. The bait used was a mixture of peanut butter, honey, and oats. The bait was positioned within the modified focal range of a browning BTC-7-4K camera [34]. Selfie traps were rebaited and resprayed with honey water halfway into the 28-night survey sessions. 

Camera trap footage collected was sorted for each site to species, and individual where possible. Individuals were identified from either: (1) ear notches made when animals were caught from live trapping; (2) unique distinguishable natural scars on ears; or (3) a combination of facial markings, tail tip colour (in the case of sugar gliders) or other unique feature (e.g., presence of tick, or tail injury). Profiles were created for each individual animal to aid in identification, and where a video could not be confidently assigned to an individual it was assigned as an ‘unknown individual’ and not used. 

The sex of each individual was recorded where possible. For sugar gliders, males were readily identified by the presence of a bald patch on their foreheads, as well as scent glands on their chest. Their testicles were also often easily observed on camera as well. Female gliders were identified only if the presence of a pouch was observed, as the absence of a bald patch could indicate a sub-adult male. Male antechinus were identified by the presence of testicles and female antechinus by the presence of a pouch or pouch young. Rattus species were identified as male if testicles were observed, and females were identified by a clear lack of testicles as adults. 

### 2.4. Analysis

Recapture rates were firstly calculated at a nightly interval (i.e., independence criterion of 24 hrs between an observation at a site). Recapture rates were summarised per species, per area, and observations included sightings of individuals across multiple sites in a single night. However, the final dataset used observations separated by ten minutes (lowest common interval across all areas), as some individuals were observed moving between adjacent cameras as quickly as four minutes apart. From this final dataset, total movements of individual small mammals were calculated using the Tracking Analyst tool in ArcGIS 10.7.1 (Esri, Redlands, CA, USA) [50]. 

Minimum convex polygons (MCP) are a simple and frequently used method for estimating home range size however it is highly sensitive to sample size, outliers and assumes an even distribution, and can often lead to over-estimation of home range [51]. In this study, most individual animals were only recorded at less than three locations. Therefore, MCPs were performed and masked by available habitat to visualise areas of habitat being used by individuals but were not used in formal analyses. Masking was performed by using the Extract by Mask tool in ArcGIS [50] to highlight the useable habitat occurring within each MCP and remove unusable habitat (cleared areas like pastures or built environments) from the MCP.

Due to the non-normal nature of the recapture data, non-parametric Mann-Whitney U-test were used to compare distances moved by male and female antechinus and sugar gliders, in linear strips and in fragments of habitat.

For the target species, kernel density estimates (KDE) of 95% and 50% home ranges, were performed for sugar gliders that were observed at more than four sites (n = 34), using the R package ‘LatticeDensity’ [52] in R Studio (RStudio, PBC, Boston, MA, USA) [53]. ‘LatticeDensity’ was used to estimate KDE as it allows for home range analyses to account for irregular boundaries of habitat, and gaps in habitat, which is a critical consideration for the study landscape where the available habitat is fragmented at fine scales. As recapture rates (per 24 h) ranged between 6 and 25 per individual, to determine the impact of sample sizes on KDE home ranges, regression analysis were performed using JMP Pro 15 (SAS Institute Inc.: Cary, NC, USA) [54]. As there was no significant relationship between recapture rates and all three home range estimates (95% KDE, 50% KDE and MCP), then all 34 individual sugar gliders were included in subsequent KDE home range analyses. Due the shorter time frames of the surveys conducted, only core home ranges of 50% were used to compare differences between sex and fragmentation classes. Differences in core home range sizes between male and female gliders, and gliders within larger fragments and linear fragments, were compared using *t*-tests (assuming unequal variances) on log-transformed data (where tests for normality were not met). 

## 3. Results

### 3.1. Recaptures 

Across all four areas, selfie traps recorded 28,566 videos of animals, across 4592 camera trap nights. Of the videos, 53.3% of videos were assigned to an individual and the remainder as unknown individuals. A total of 284 small mammals were individually identified. Approximately, 56% of these individuals had movements recorded between sites (i.e., observed at two or more sites; see Table S1 in Supplementary Materials). As selfie traps were placed on planks positioned up trees, the species that was most recorded on camera was the arboreal sugar glider and semi-arboreal brown antechinus (Table 1 and Table S1). Across all 284 small mammal individuals, 92% were able to be sexed (females: 52%, males: 40%). 

Capture success was high for sugar gliders and antechinus in most areas surveyed (Table 1). Area four had the least recapture rate for gliders, and area two had the lowest rate of recaptures for antechinus. Both *Rattus* species had low capture success across all areas (Table 1). 

At the individual level, female sugar gliders were captured on camera on an average of 11.2 (±0.6) nights (maximum of 20) and male sugar gliders were captured 9.0 (±0.6) times (maximum of 23). In comparison, the highest recapture rate for an individual glider during one week of live trapping in this study was three. Female antechinus were caught on cameras an average of 14.9 (±1.5) times (maximum of 26), and male antechinus were captured at an average of 6.4 (±1.2) times (maximum of 20). The highest live capture rate for an individual antechinus was four. Only a small number of individual rats were recaptured on camera, and these recaptures often occurred only at one site (Table 2 and Table S1). 

### 3.2. Movement: Straight Line Distances

**Figure 4:**
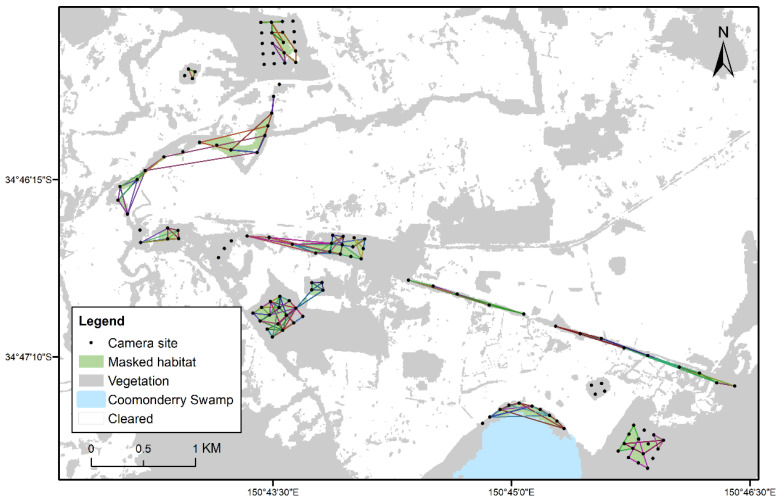
Polygons formed for 121 sugar gliders (*Petaurus breviceps*) where individuals (females = 63, males = 58) were observed on at least two selfie trap sites. Useable habitat within polygons (masked habitat) is highlighted in green.

**Figure 5:**
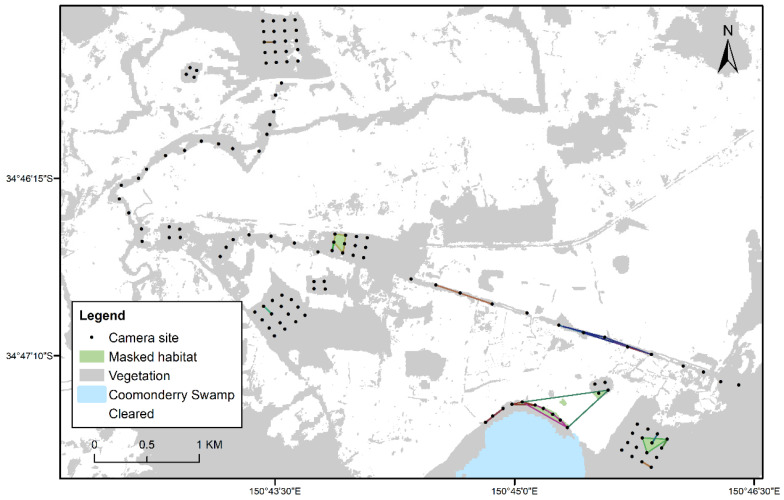
Polygons formed by connected sites for 20 brown antechinus (*Antechinus stuartii*) where individuals (females = 9, males = 11) were observed on at least two selfie trap sites. Useable habitat within polygons (masked habitat) is highlighted in green.

Within a four-week survey period, the maximum distance travelled by an individual glider as detected by camera traps, was 14,661 m (Table 3). The maximum distance observed for an individual brown antechinus was 4237 metres (Table 3). Distances moved by sugar gliders within a month ranged between 0 to 14,661 m, with averages between 1013 m for females and 1167 m for males (Table 3). Distances moved by antechinus ranged from 0 m to 4237 m, with averages between 243 m for females and 861 m for males (Table 3). Most movements recorded were within each sampling unit, either within a grid or within a linear transect (Figure 4 and Figure 5). For both rat species, there were insufficient observations made due to the spacing and positioning of the selfie traps up trees. However, one male bush rat travelled 1902 m, and a male brown rat was observed travelling 1283 m (Table 3). 

Within a single night, the most distance moved for a female sugar glider was 1411 m and for a male glider, it was 1760 m. One female antechinus was observed moving 880 m in one night, and a male antechinus moved 1080 m. 

### 3.3. Movements within and between Fragments of Habitat

Across 121 sugar gliders, the amount of useable habitat within MCPs for an individual ranged from 3.1 ha to 5.2 ha (Figure 4). Gliders were detected in all sampled fragments and areas, except for one isolated patch surrounded by cleared land (see north-east of Coomonderry Swamp; Figure 4). Across 20 antechinus, the amount of useable habitat within a MCP for an individual ranged from 1.3 ha to 3.1 ha (Figure 5). Antechinus were detected in all areas, however the wide spacing of trap sites meant that most movement observations were restricted to less than two sites and only 29% (21/72 individuals) were recorded at two or more sites (Table S1). 

Nine between fragment movements (movements made between distinct grids and linear transects, or between large gaps in forest cover) were recorded for three individuals. Though actual movement pathways remain unknown, it is likely that animals would have to have travelled through cleared areas as there was little or no connectivity between patches (Figure 4 and Figure 5). Two sugar gliders were recorded moving between large gaps in canopy, one crossed this gap (43m) on six occasions, and the other crossed another gap (39m) once (Figure 4). One antechinus was recorded moving between the swamp edge and an isolated habitat patch, across cleared empty paddocks, with straight line distances of 1359m (Figure 5). 

**Figure 6:**
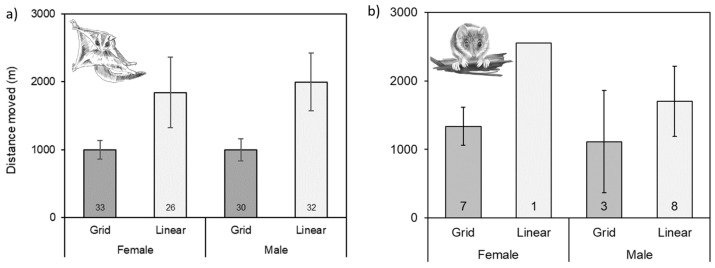
Mean distances moved (±standard error) between recaptures within patches of habitat (sampled in grids) and in linear strips (sampled as transects) for female and male (**a**) sugar gliders (*Petaurus breviceps*); and (**b**) brown antechinus (*Antechinus stuartii*). Individuals used in analysis were detected at two or more sites. Numbers below bars represent N sample size. Credit for illustration on left: Rocco Russo; and on right: Tracy MacVean.

For female sugar gliders, mean distances moved between recaptures in patches and linear strips, were not significantly different (Mann Whitney U = 1062.50, P = 0.304; Figure 6a). For male sugar gliders, mean distances moved were significantly greatest in linear strips compared to forest patches (U = 952.00, P < 0.05; Figure 6a).

Distances moved by female antechinus in forest patches and linear strips, were not significantly different (U = 289.00, P = 0.87; Figure 6b). For male antechinus, mean distances moved were greatest in linear strips compared to forest patches, though differences were not significant (U = 59.00, P = 0.10; Figure 6b). 

### 3.4. Home ranges & Social Organisation of Sugar Gliders

Short term core (50% KDE) homes ranges of 34 sugar gliders (detected at four or more sites) ranged from 0.4 ha to 4.3 ha (see Table S2 in Supplementary Materials for all estimates). Sugar glider core home ranges were on average 1.2 ha (±0.2) for all females and 2.4 ha (±0.3) for all males. In fragments of habitat measured in grids, core home ranges were 1.1 ha females and 1.4 ha for males (Table 4). In linear fragments of habitat, core home ranges 1.2 ha for females and 2.7 ha for males (Table 4). The size of core home ranges did not significantly differ between female and male sugar gliders (t = 1.37, df = 28.42, P = 0.09), nor between gliders within larger fragments sampled as grids, and linear forms of habitat (t = 1.27, df = 23.96, P = 0.21). 

Of the core home ranges calculated, there were 21 instances of home range overlap. Of these overlapping home ranges, 67% were between males and females, 19% occurring between females, and 14% between males. The average percentage overlap for sugar gliders of male-female was 14% (±4%), male-male 20% (±9%) and for female-female 20% (±8%). The maximum overlap between two females was 38% (1.7 ha); two males was 25% (0.5 ha), and between a female and a male was 55% (0.5 ha).

There were 313 events where two or more gliders were observed in the selfie trap together, where all individuals were identifiable in the video. Of these co-occurring individuals, 50% (155) of observations were two females, 42% (133) were a female and male pair, 6% (20) were two females and one male, and the remaining 2% (5) were a pair of males.

## 4. Discussion

### 4.1. Recapture Rates

The rates of recapture for individual sugar gliders obtained in this study can be considered high. Studies capturing sugar gliders using live traps have had varied success rates, ranging from 5.1% [55], 8.2% [56], 9.7% [47] and 27.7% [45]. Through camera trapping our study achieved high rates of capture (up to 70%) as selfie traps act as an ‘open’ trap which allows multiple individuals of different species to be detected each night, across sites [39]. In addition to this, the use of bait attracts animals to return, however the bait was enclosed with limited access to ensure continued visitation during the four-week surveys. Though bait impacts on animal behaviour, an individual must first be ranging in the area to locate the selfie trap. In our study, when baits began to run low, many visits were quick. Gliders spent only a few seconds inside the selfie trap to smell the bait holder, before immediately leaving (there was an average decline of 30.5% in the number of videos recorded in the last three days of a session, when compared to the first three days since baited). Thus, for long term home range studies using selfie traps we recommend that baiting occurs irregularly and with randomisation using olfactory lures (e.g., cotton wool soaked with vanilla essence [42]). This is so that animals do not learn to respond to a consistently scheduled bait source but rather that selfie traps detect their within-area movements. 

The recapture rates for individual antechinus can also be considered high, when compared to published rates of recapture for similar species. For example, one study obtained an average recapture rate of 4.3 individual brown antechinus (males and females) across 16,630 live trap nights [57]. Comparatively, our study was able to obtain high rates of recaptures per unit effort when using selfie traps (average nightly recapture rates for antechinus was 11.1, across 28 trap nights). Thus, the selfie trap method is useful for mark-resight methods for small mammals due to the high rates of recapture that can be obtained. 

### 4.2. Movement: Straight Line Distances

The maximum distances moved as observed on selfie traps, were high for both sugar gliders and antechinus. Few studies report total or nightly movements made by individual gliders, and this is likely due to observations being made via radio-tracking and not through GPS-collars. The nightly movements as observed through selfie traps, made by both antechinus and sugar gliders, indicates high mobility and an ability to use linear forms of habitat despite significant edge effects. For Antechinus, the most amount of movement we observed for an individual for one night was 880 m for a female, and 1080 m for a male. Comparatively, a live trapping study by Marchesan and Carthew (2004) detected one male traversing 540 m in a single night (*Antechinus flavipes*). 

For sugar gliders, the most distance we observed in a single night was 1411 m (female) and 1760 m (male). Comparatively in one other study, a male sugar glider was reported to move 360 m in one night [58]. Other congenera species have detected large nightly movements of 1561 m in male *Petaurus norflocensis* [59], and 3430 m of movement in male *Petaurus gracilis* [60]. For female *P. norflocensis* 1909 m of movement in a single night has been previously recorded [59]. Our study was able to observe large movements of gliders, both at the nightly scale and across the whole study duration. This indicates that the selfie trap method could be readily used in movement ecology study for a range of glider species, such as to look at the impacts of various disturbances (e.g., roads, agriculture, urbanisation) on glider movements. 

### 4.3. Movements within and between Fragments of Habitat

Our study identified native small mammal persistence in very small patches of habitat and along linear roadsides, surrounded by highly disturbed agricultural lands. Though most movement observations were detected within fragments, sugar gliders were detected travelling across canopy gaps (presumably by gliding). Sugar gliders can persist in highly fragmented landscapes and move through the matrix where structures are available as steppingstones to permit gliding [58,61,62]. In comparison, the ability of one antechinus to move through a large open paddock indicates the species can traverse through the matrix. Similar observations were made for *A. flavipes* where 17 (4% of all movements) between fragment movements were recorded [63]. Our findings contribute to the idea that both brown antechinus and sugar gliders have some matrix tolerance for dispersal, which is likely an important behavioural advantage to their persistence in fragmented landscapes [9,58,64,65,66]. 

In another study, foraging distances of the mahogany glider per night ranged from 590–3430 m [60]. Foraging distances varied throughout the year and this was related to the flowering index in the study landscape, indicating changes in behaviour in response to dynamic food resources in the area [60]. In our fragmented areas, nearly all habitat was predominantly *Eucalyptus* forest, though creek lines and swamps were dominated by *Casuarina glauca.* Interestingly all gliders in our study that were detected in Coomonderry swamp, were not detected in the nearby forest. Gliders when released were observed gliding west, away from the closest *Eucalyptus* forest. Furthermore, on one occasion a glider when released from live trapping was observed gliding onto a dead *C. glauca* and entering a hollow. These observations could indicate glider residency within a swamp. The canopy edge was incredibly narrow (five metres wide), dominated by Casuarina trees, with only a few *Banksia serrata* present (not in flower during the survey period) and most available vegetation was at a height of less than two metres (thickets of *Melaleuca* scrub). The residency of sugar gliders within the swamp may be explained by possible access to other areas, perhaps at different times of year to access associated changes in flowering plants. However, our live trapping and camera trapping does data suggests significant use of a swamp by sugar gliders. Other studies have documented the use of low strata vegetation by sugar gliders, this being in banksia heaths, with patches of woodland nearby [67,68]. 

The use of space and home range will vary between individuals of species, because of the distribution of resources (food, shelter, mates) within an area [69,70,71]. Where habitat patch size is small, behavioural adaptions to obtain resources can occur, such as brushtail possums will range further in linear strips of habitat [13]. In our study, within larger fragments of habitat, less movements were made by individual antechinus and sugar gliders, compared to conspecifics found within linear forms of habitat (creek lines and roadsides). However, sample sizes were small and variability high, thus differences were not significant between females in patches of habitat and linear habitats. Male movements were higher in linear habitat forms, and this could be partly attributed to the dispersal of male antechinus and sugar gliders during the breeding season as they search for mates. Other studies identified changes in home range size and shape due to linearity of habitat available [72,73,74]. One resource such as hollows (den sites) may be a limiting factor in the study landscape. As part of a separate study, surveys into hollow availability were conducted in various locations in this study’s landscape. The surveys found the availability of hollows were limited in various fragments and linear strips of habitat. 

Overall, the limitation of the movement data from this study was that survey periods were relatively short, and that whole patches of habitat could not always be surveyed entirely (limited by access to private property), thus many gliders that were observed at one or two sites may only reflect the edge of their home range. 

### 4.4. Home Ranges & Social Organisation of Sugar Gliders

Core home ranges of sugar gliders in our fragmented landscape were similar to that of other studies, despite potential methodological differences [40,56,58]. Our study found core home ranges (50% KDE) ranged from 0.35 ha to 4.26 ha, with an average of 1.55 ha (across both females and males). In a continuous forest, Quin [56] found sugar glider short-term home ranges ranged from 0.79 to 1.75 ha, with an average of 1.27 ha (added squares method), with no differences found between seasons and sex. In another continuous forest, sugar glider home ranges were on average 5–6 ha (radio tracking) [40]. However in a fragmented system of forest, home ranges were smaller with an average of 0.6 ha (manually mapped) [58]. Compared to these studies, our home range estimates may reflect an overall response to the level of fragmentation within the study landscape. Species can either restrict their home ranges, or be forced to range further, depending on the distribution of resources in a fragmented or disturbed system [75]. At a fine scale, our study found no differences in core home range sizes between fragment type. However, long-term study would be needed to directly compare large continuous forests (i.e., Illawarra Escarpment to the North and Seven Mile Beach National Park to the South), with the fragmented areas located centrally in the study area, to determine if there is a fragmentation effect on home range size. 

In our study, 95% KDE and MCP home ranges, reached estimates of 16.44 ha and 5.2 ha respectively. Though these are overestimates of home range, it allows us to identify that some individuals were exhibiting large ranging behaviours (see Figure 4). These observations could be explained by male gliders dispersing [9,58,76] or ranging behaviour in response to food resources changing with season and time (e.g., Savannah glider, *Petaurus ariel*; [60,69]). Studies into gliding species have found that home ranges increase in size in fragmented or urban areas, which has been attributed to the stretched and disjointed distribution of resources in the landscape [77,78]. In our study, some of the wide-ranging movements observed may be further explained by the limited amount of habitat and its connectivity. For the savannah glider, it was found that when resources were limiting, home-range size can far exceed what is predicted by body mass and diet [69]. However, Jackson [60] found that the mahogany glider (*Petaurus gracilis*) had smaller home ranges in fragmented habitat (6–11 ha) than in continuous habitat (19–20 ha), and the reasons for this were unclear though the authors suggested that home ranges in fragmented landscapes are unstable. 

The overlap in core home ranges in our study are reflective of the species social organisation, where groups are made of one or two codominant males and the remaining family group consists of females [56,58]. Family groups have one or two dominant males that passively mark territory with their scent glands [79], and will also scent mark individuals in their group [80]. Groups are territorial and do not socialise [80], thus the number of different individuals overlapping at sites is likely to indicate the different groups occurring at that site. In our study gliders were recorded on camera in pairs, and on multiple occasions groups of three and four gliders were seen on camera. The proportions of co-occurring individuals on camera confirm observations as seen by other authors, where female-female and female-male co-occurrence are more common than male-male co-occurrence [58,76]. We observed on several occasions male-male glider antagonistic behaviour, where a male approaching another male glider already inside the selfie trap would be met with aggressive snarls and grunts, and in some cases, chased away. Scent marking behaviour was also observed where male gliders rubbed their chests along the entrance of selfie traps. Gliders could be observed often sniffing the air upon approaching the selfie trap, and on some occasions, this was followed by males immediately leaving on their first visit.

Sugar gliders form colonies that can range up to 12 individuals [58]. In our study, there was one occurrence where 12 individuals were detected at a single selfie trap, indicating the potential presence of a large family group in our study landscape, occurring in a linear strip of habitat. However, there is the possibility that some of these individuals may have been transient or at the edge of their home range given that four of the males were only observed on one to three independent nights. 

## 5. Conclusions

The selfie trap is proven to be an effective camera trapping method for detecting movements and behaviour of small mammals. Due to the high rates of recapture the method can be readily applied to long-term studies that estimate home ranges. We found that linear forms of habitat and larger fragments throughout the study landscape provided both habitat and pathways for movement. These linear strips along roadsides and creek lines provide some of the last remaining habitat corridors for both arboreal and ground dwelling small mammals. Efforts must be made to conserve and rehabilitate these areas through additional plantings (especially feed trees) and tree hollow conservation and supplementation (nest box or artificial cavity installation). 

## Figures and Tables

**Table 1:** Total number of independent observations (separated by 24 h) made for each small mammal. Observations include where individuals were recorded at different sites during a single night. The current study performed 4592 camera trap nights in total, with each area (thus each individual) camera trapped for 28 nights. Capture success was calculated by diving the total number of independent observations made per species, in each area, by camera trap nights. The number of sites in each area was: Area one = 44, Area two = 45, Area three = 40, and Area four = 40.

	Female	Male	Unknown Sex	Total	Capture Success
Area 1					
*Petaurus breviceps*	265	251	15	531	43%
*Antechinus stuartii*	40	115	9	164	13%
*Rattus fuscipes*	0	2	0	2	0%
*Rattus norvegicus*	4	22	1	27	2%
Area 2					
*Petaurus breviceps*	529	351	5	885	70%
*Antechinus stuartii*	248	14	15	277	2%
*Rattus fuscipes*	18	48	1	67	1%
*Rattus norvegicus*	6	0	0	6	1%
Area 3					
*Petaurus breviceps*	172	108	41	321	29%
*Antechinus stuartii*	34	0	0	34	3%
*Rattus norvegicus*	15	16	8	39	3%
Area 4					
*Petaurus breviceps*	11	1	0	12	1%
*Antechinus stuartii*	306	0	0	306	27%
*Rattus fuscipes*	35	0	0	35	3%
*Rattus norvegicus*	20	13	0	33	3%

**Table 2:** Average rates of selfie trap recaptures for each individual (for those confidently sexed). Recaptures refer to the number of independent nightly observations (i.e. observations separated by 24 h), and includes observations where individuals were recorded at different sites during a single night. The current study performed 4592 camera trap nights in total, with each area (thus each individual) camera trapped for 28 nights.

	N	Mean Recapture Rate	S.E.
*Petaurus breviceps*			
Female	87	11.2	0.6
Male	77	9.0	0.6
*Antechinus stuartii*			
Female	49	14.9	1.5
Male	21	6.4	1.2
*Rattus fuscipes*			
Female	4	14.8	5.0
Male	7	8.0	2.5
*Rattus norvegicus*			
Female	9	6.3	1.4
Male	8	7.0	1.4

**Table 3:** Movement distances observed on selfie traps for individual small mammals (of known sex) over 28-trap night periods, between August and November 2019. Included are the mean and maximum of direct straight-line distances moved by an individual small mammal.

	N	Mean Number of Sites Detected at	S.E.	Mean Distance Travelled (m)	S.E.	Maximum Distance Observed (m)
*Petaurus breviceps*						
Female	87	2.41	0.13	1013	200	14,661
Male	77	2.68	0.17	1167	205	12,015
*Antechinus stuartii*						
Female	49	1.27	0.09	243	91	2555
Male	21	2.19	0.32	861	292	4237
*Rattus fuscipes*						
Female	4	1.00	0.00	0	0	0
Male	7	1.57	0.20	394	264	1902
*Rattus norvegicus*						
Female	9	1.00	0.00	0	0	0
Male	8	1.38	0.18	344	190	1283

**Table 4:** Short term home ranges (50% and 95%) using Kernel Density Estimate (KDE), of sugar gliders (*Petaurus breviceps*) detected at four or more sites (n = 34). Recapture rates were calculated as the number of distinct nights and site, that an individual was observed at. Minimum convex polygon (MCP) home range estimates were calculated by masking available habitat. Standard Error is show in brackets.

	N	Number of Sites Detected at	Recapture Rate	Distance Moved (m)	KDE Home Range 95% (ha)	KDE Home Range 50% (ha)	MCP Home Range (ha)
**Grid**							
Female	9	4.3 (0.2)	15.0 (1.8)	1697 (224)	4.6 (0.4)	1.1 (0.1)	1.3 (0.1)
Male	8	4.6 (0.3)	11.3 (1.7)	1503 (328)	6.9 (0.9)	1.4 (0.3)	2.0 (0.4)
**Linear**							
Female	6	4.7 (0.3)	18.2 (2.8)	5027 (2208)	5.6 (1.4)	1.5 (0.4)	1.2 (0.2)
Male	11	4.8 (0.4)	11.8 (1.7)	4000 (954)	7.5 (1.2)	2.1 (0.4)	2.7 (0.5)

## Data Availability

The datasets generated during and/or analysed during the current study are available from the corresponding author on reasonable request.

## Supplementary Materials

The following supporting information can be downloaded at: https://www.mdpi.com/article/10.3390/ani12070912/s1, Table S1: Number of individual small mammals detected at various spatial samples on selfie traps positioned in the four areas within the study landscape; Table S2: Short term home ranges (50% and 95%) using Kernel Density Estimate (KDE), of sugar gliders (*Petaurus breviceps*) detected at four or more sites (n = 34). Female n = 15, male n = 19. Home ranges estimates from Minimum Convex Polygons (MCP) was calculated by calculating the amount of available habitat within the polygon.
