# Supplementary material for: The Use of Selfie Camera Traps to Estimate Home Range and Movement Patterns of Small Mammals in a Fragmented Landscape
Source: Animals (Basel). 2022 Apr 2;12(7):912. doi: 10.3390/ani12070912 (PMC8997104; doi:10.3390/ani12070912)
Supplement: Supplementary file 1 [file animals-12-00912-s001.zip › animals-1649677-supplementary.pdf]

**Table S1.** Number of individual small mammals detected at various spatial samples on selfie traps positioned in the four areas within the study landscape.

| | Number of individuals detected at only | | | | | | | |
|---|---|---|---|---|---|---|---|---|
| | **1 site** | **2 sites** | **3 sites** | **4 sites** | **5 sites** | **6 sites** | **8 sites** | **Total** |
| **Area 1** | | | | | | | | |
| *Petaurus breviceps* | 14 | 23 | 12 | 4 | 3 | 2 | 1 | 59 |
| *Antechinus stuartii* | 15 | 5 | 3 | 4 | | 1 | | 28 |
| *Rattus fuscipes* | 1 | | | | | | | 1 |
| *Rattus norvegicus* | 7 | | | | | | | 7 |
| **Area 2** | | | | | | | | |
| *P. breviceps* | 23 | 21 | 22 | 12 | 4 | 1 | | 83 |
| *A. stuartii* | 20 | 4 | | 1 | | | | 25 |
| *R. fuscipes* | 5 | 4 | | | | | | 9 |
| *R. norvegicus* | 1 | | | | | | | 1 |
| **Area 3** | | | | | | | | |
| *P. breviceps* | 7 | 9 | 8 | 5 | 2 | 1 | | 32 |
| *A. stuartii* | 1 | 1 | | | | | | 2 |
| *R. norvegicus* | 5 | 3 | | | | | | 8 |
| **Area 4** | | | | | | | | |
| *P. breviceps* | 4 | | | | | | | 4 |
| *A. stuartii* | 15 | 1 | 1 | | | | | 17 |
| *R. fuscipes* | 2 | | | | | | | 2 |
| *R. norvegicus* | 5 | 1 | | | | | | 6 |
| **Total** | 125 | 72 | 46 | 26 | 9 | 5 | 1 | 284 |

**Table S2.** Short term home ranges (50% and 95%) using Kernel Density Estimate (KDE), of sugar gliders (*Petaurus breviceps*) detected at four or more sites (n = 34). Female n = 15, male n = 19. Home ranges estimates from Minimum Convex Polygons (MCP) was calculated by calculating the amount of available habitat within the polygon.

| Individual no. | Area | Fragment | Sex | Number of sites detected at | Recaptures (/24hr) | Distance moved (m) | KDE Home range 95% (ha) | KDE Home range 50% (ha) | MCP (ha) |
|---|---|---|---|---|---|---|---|---|---|
| 1 | A1 | Grid | F | 5 | 10 | 2425.44 | 5.69 | 1.26 | 2.12 |
| 2 | A1 | Grid | M | 4 | 9 | 702.58 | 3.94 | 0.61 | 2.14 |
| 3 | A3 | Linear | M | 5 | 10 | 1479.05 | 3.71 | 1.00 | 1.77 |
| 4 | A3 | Linear | F | 5 | 14 | 2397.75 | 3.33 | 0.80 | 1.77 |
| 5 | A3 | Linear | M | 4 | 6 | 2378.03 | 8.97 | 1.60 | 5.20 |
| 6 | A3 | Linear | M | 4 | 13 | 4438.07 | 5.77 | 1.29 | 4.40 |
| 7 | A3 | Linear | M | 4 | 14 | 3474.96 | 12.52 | 3.95 | 3.58 |
| 8 | A3 | Linear | F | 4 | 20 | 1791.58 | 3.39 | 0.92 | 1.06 |
| 9 | A3 | Grid | M | 6 | 9 | 2325.24 | 7.60 | 1.40 | 2.20 |
| 10 | A1 | Linear | M | 5 | 9 | 7145.37 | 16.44 | 4.26 | 1.19 |
| 11 | A1 | Linear | M | 4 | 9 | 1866.25 | 5.20 | 1.01 | 0.70 |
| 12 | A1 | Linear | M | 4 | 7 | 928.26 | 8.09 | 3.83 | 0.94 |
| 13 | A1 | Linear | F | 4 | 7 | 763.94 | 11.95 | 2.43 | 0.34 |
| 14 | A1 | Linear | M | 8 | 25 | 12014.52 | 7.72 | 3.08 | 4.48 |
| 15 | A1 | Linear | M | 6 | 18 | 4276.08 | 3.21 | 1.42 | 3.75 |
| 16 | A1 | Linear | F | 6 | 27 | 14660.74 | 6.23 | 2.07 | 1.45 |
| 17 | A1 | Linear | F | 5 | 22 | 8252.85 | 5.71 | 2.32 | 1.26 |
| 18 | A2 | Grid | M | 6 | 20 | 3122.04 | 5.83 | 0.81 | 3.59 |
| 19 | A2 | Grid | M | 5 | 10 | 643.31 | 11.29 | 2.94 | 2.80 |

| | | | | | | | | | |
|---|---|---|---|---|---|---|---|---|---|
| 20 | A2 | Grid | F | 5 | 16 | 1599.44 | 5.40 | 1.51 | 1.56 |
| 21 | A2 | Grid | M | 4 | 7 | 1342.98 | 10.16 | 2.44 | 2.97 |
| 22 | A2 | Grid | M | 4 | 17 | 2048.99 | 6.84 | 1.45 | 0.05 |
| 23 | A2 | Grid | F | 4 | 20 | 1306.54 | 3.13 | 0.55 | 1.42 |
| 24 | A2 | Grid | F | 4 | 9 | 1444.40 | 4.17 | 1.08 | 1.05 |
| 25 | A2 | Grid | F | 4 | 10 | 1342.20 | 4.65 | 0.84 | 1.14 |
| 26 | A2 | Grid | F | 4 | 14 | 2167.65 | 5.57 | 1.71 | 1.02 |
| 27 | A2 | Grid | F | 5 | 21 | 1297.86 | 4.83 | 0.91 | 1.31 |
| 28 | A2 | Grid | M | 4 | 6 | 515.08 | 4.40 | 1.09 | 1.29 |
| 29 | A2 | Linear | M | 5 | 8 | 2559.74 | 4.01 | 0.67 | 2.47 |
| 30 | A2 | Linear | M | 4 | 11 | 3437.96 | 6.45 | 0.52 | 1.35 |
| 31 | A2 | Linear | F | 4 | 19 | 2294.83 | 2.78 | 0.35 | 1.43 |
| 32 | A2 | Grid | M | 4 | 12 | 1323.24 | 5.03 | 0.69 | 1.28 |
| 33 | A2 | Grid | F | 4 | 23 | 771.67 | 2.26 | 0.56 | 0.92 |
| 34 | A2 | Grid | F | 4 | 12 | 2914.36 | 6.01 | 1.32 | 1.14 |
